# Study on Soil Selenium-Induced Copper Deficiency in Yudong Black Goats

**DOI:** 10.3390/ani14101481

**Published:** 2024-05-16

**Authors:** Guangyang Liu, Xiaoyun Shen

**Affiliations:** School of Life Science and Engineering, Southwest University of Science and Technology, Mianyang 621010, China; guangyangliu@mails.swust.edu.cn

**Keywords:** soil, forage, trace element, immunity, antioxidative status

## Abstract

**Simple Summary:**

The aim of this study was to find out the cause of copper deficiency in the Yudong black goats by investigating the mineral content of their habitat. We found that the root cause of this phenomenon was selenium deficiency in the habitat soil and then discovered ways to alleviate this symptom. This is extremely beneficial for further research on copper deficiency diseases in Yudong black goats.

**Abstract:**

Due to the degradation of pasture and strict restrictions on grazing ranges in recent years, copper (Cu) deficiency in Yudong black goats has been occurring, mainly manifested as emaciation, anemia, loss of appetite and lack of spirit. To explore the main causes of Cu deficiency in Yudong black goats, 40 black goats (1 year old, 25.11 ± 0.52 kg) were selected for this experiment; among them, 20 Yudong black goats with Cu deficiency from the experimental pasture were used as the experimental group, and 20 healthy Yudong black goats from the control pasture were used as the control group. In the pre-experiment, the mineral contents of the soil, forage, blood, and liver of black goats in both groups were determined, and in formal experiments, blood hematological, biochemical, antioxidant, and hemorheological parameters were analyzed. An experiment on the treatment of Cu deficiency in black goats was also conducted. This study showed that selenium (Se) levels in the soil, forage, blood, and liver from the experimental group were significantly lower than those from the control group (*p* < 0.01). The content of sulfur (S) in the forage was considerably higher than that of the control group (*p* < 0.01). The contents of Cu in the blood and liver from the experimental group were significantly lower than that from the control group (*p* < 0.01), and the content of S was considerably higher than that from the control group (*p* < 0.01). The blood hematology of the experimental group was affected, as evidenced by a decrease in hemoglobin, hematocrit value, mean corpuscular volume and mean corpuscular hemoglobin. The immunity and antioxidant capacity of black goats in the experiment group were impaired to varying degrees, with significant decreases in ceruloplasmin, immunoglobulin M, immunoglobulin G, glutathione peroxidase, and superoxide dismutase, and substantial increases in malondialdehyde. In addition, the experimental group showed a decrease in blood viscosity as evidenced by the rise in high shear viscosity, low shear viscosity, erythrocyte rigidity index, erythrocyte aggregation index, and erythrocyte deformation index, and a decrease in plasma viscosity. In the treatment experiment, oral administration of copper sulfate solution was carried out on 10 black goats with Cu deficiency. All the Cu deficiency goats were cured, and the Cu content in their bodies rebounded. In summary, low Se soil caused an increase in S content in the forage, and Yudong black goats feeding on high S forage resulted in a decrease in Cu absorption, which led to a secondary Cu deficiency.

## 1. Introduction

The Yudong black goat is a local breed that is kept for skin and meat and is mainly distributed in Wulong County and its surrounding areas in southwest China. In the Third National Census of Livestock and Poultry Genetic Resources in 2021, the number of Yudong black goat populations in Youyang was 9445, which were mainly distributed in more than 20 townships, such as Mawang Township, Maoba Township, and Muye Township. Yudong black goats are characterized by their uniformly black coat covering the entire body. Adult rams typically have thicker and longer hair than ewes, whose hair is shorter. They feature a triangular-shaped head of medium size with a straight nose erect and upward ears and often possess horns and whiskers. Both rams and ewes commonly exhibit these features. Their head, neck, and trunk are compactly combined, with the hindquarters slightly higher than the front. Farmers and consumers favor the Yudong black goat because of its easy management, high reproduction rate, and tender meat [1,2].

In recent years, due to pasture degradation and strict restriction of grazing range [3], the behavioral mechanism of balanced mineral nutrition in Yudong black goats has been impaired, resulting in more and more Yudong black goats suffering from copper (Cu) deficiency, which is mainly manifested as emaciation, anemia, loss of appetite, and poor mental health, which has brought significant economic losses to local farmers.

Selenium (Se) and sulfur (S) are both essential elements in wildlife and livestock, and they belong to the same family of elements that share a common uptake site in the cell wall of plant roots because of their structural similarity and have a similar affinity for the site. It has been shown that there is competition between selenate and sulfate [4], and the size of the ratio of the two elements in the environment determines the extent of their uptake by plants. Therefore, environmental Se deficiency enhances S uptake by plants and vice versa. Cu is an essential nutrient for animal growth and development [5]. Many of the world’s Cu-deficient pastures are unsuitable for grazing, and according to a UK analysis of serum Cu levels in ruminants, a number of diseases have been associated with Cu deficiency, such as anemia, ataxia, wasting, etc. The annual incidence of Cu deficiency was about 0.9% of the total. In Cu-deficient areas, ataxia in lambs can be as high as 30% in some areas. Although deaths were rare, sheep failed to thrive, resulting in severe economic losses [6,7,8]. Cu deficiency includes direct and induced deficiencies. When ruminants have S and molybdenum (Mo) in the rumen, Cu will form poorly absorbed Cu-S-molybdate complexes with S and Mo. Then, induced Cu deficiency will occur. Even high S intake can also greatly reduce Cu absorption, which does not depend on Mo [9,10]. When the Cu content of the forage is significantly lower than the healthy level, direct Cu deficiency will occur. Most of the Cu in the serum exists in the form of ceruloplasmin (Cp), which can increase the iron (Fe) saturation in ferritin and promote the transport, absorption and utilization of Fe by bones, so Cu deficiency will further lead to the decline of Cp content, which will lead to Fe-deficiency anemia [11]. In China, the solution to the problem of Cu deficiency in goat farming is mostly copper sulfate (CuSO_4_) supplementation for goats.

Hence, the Cu deficiency in Yudong black goats is likely a secondary Cu deficiency caused by Se deficiency. This study aimed to confirm this relationship, offering theoretical insights for preventing and managing Cu deficiency in these goats. Additionally, it aimed to address breeding challenges among local farmers, thereby minimizing economic losses.

## 2. Materials and Methods

### 2.1. Study Region

The study site is located in Muye Township, Youyang County, Chongqing Municipality, in the Wuling Mountains (29°6′–30°15′ N, 108°59′–107°54′ E), which belongs to the typical mountainous farming and animal husbandry counties and goat production counties in China. The experimental site belongs to the subtropical monsoon climate with a poster height of 263–1895 m, a frost-free period of 286 days, terrain undulation, average annual temperature 13 °C, abundant rainfall throughout the year, average annual precipitation of 1400 mm, with rainfall concentrated in June to August [12]. The main plant types are white fescue, Arctostaphylos, wild kudzu, plantain, fireweed, arrowroot, and sumac.

### 2.2. Experimental Design

The experimental period was 30 days and consisted of a pre-experiment and a formal experiment. In the pre-experiment, the mineral contents of the soil, forage, blood, and liver of black goats were determined in different environments. In the formal experiment, the blood hematological, biochemical parameters, antioxidant parameters, and hemorheological parameters were analyzed. CuSO_4_ solution was fed to Cu-deficient black goats to observe the therapeutic effect of CuSO_4_ on Cu-deficient black goats. The experimental plan was approved by the Institutional Animal Care and Utilization Committee of Southwest University of Science and Technology (SWUST2023023).

Experimental pastures: Zhangjiaping pasture, which is located in Zhangjiaping, Muye Township and has a high prevalence of Cu deficiency in Yudong black goats, was used as an experimental pasture, and on the other hand, Maoba pasture, which is located in Maoba, Muye Township, and does not have the phenomenon of Cu deficiency in the Yudong black goats, was used as a control pasture. Both pastures are approximately 50 hectares.

Experimental animals: The experiment included 40 black goats (1 year old, 25.11 ± 0.52 kg); among them, 20 Yudong black goats with obvious symptoms (emaciation, anemia, loss of appetite and poor mental health) of Cu deficiency in the experimental pasture were used as the experimental group, and 20 healthy Yudong black goats from the control pasture were used as the control group.

Treatment experiment: Selected another 20 Yudong black goats (1 year old, 25.36 ± 0.48 kg) with obvious symptoms (emaciation, anemia, loss of appetite and poor mental health) of Cu deficiency from the experimental pasture as treatment animals and 60 mg·kg^−1^ of 1% CuSO_4_ solution were administered orally to 10 black goats for 10 d in one course of treatment with a total of two courses. The other 10 black goats did not undergo any treatment, and all 20 Yudong black goats were free to feed and drink in the experimental pasture.

### 2.3. Sample Collection

Soil samples: Ten soil samples were randomly collected from both the experimental pasture and the control pasture, sampled at depths of 0–30 cm in 1 m × 1 m quadrangles, with each sample more than 100 m apart. 

Forage sample: Ten forage samples were collected at the soil sample sampling site of each experimental and control pasture and mowed 1–2 cm above the ground to minimize soil contamination.

Blood sample: Two blood samples were collected from each Yudong black goat, for a total of 40 samples in each group. A vacuum blood collection tube containing EDTA-K_2_ anticoagulant was used to take 10 mL of blood from the jugular vein for hematology, and the anticoagulant tube was kept at 4 °C until the end of the hematology analysis. Vacuum blood collection tubes with 1% sodium heparin as an anticoagulant were used to take 10 mL of blood stored at −20 °C for trace element analysis in the pre-experiment. For biochemical analyses in the formal experiment, serum samples were collected in separating gel accelerator tubes, centrifuged at 3500× *g* for 10 min, and stored at −80 °C in EP tubes for analysis.

Liver sample: One liver sample was collected from each Yudong black goat for a total of 20 samples. Performed liver puncture biopsies on each experimental animal to extract 10–25 mg of liver tissue: before puncture biopsy, ultrasonography was performed on the black goat to observe the size and location of the liver. During the surgery, the goat was fixed lying to the left side, and then the hair on the right side of the goat’s liver position was cleanly removed. Then, the exposed skin was sterilized with alcohol, the firing button of the biopsy gun was adjusted, and the needle was inserted into the skin vertically on the abdominal wall from the penultimate 4th to 5th intercostal space intersecting with the level of the shoulder joints. The needle was kept at a 45-degree angle to the abdominal wall until the sensation of a breakthrough appeared, and at this time, the needle had already entered the liver. The launch button was gently pushed to the front of the needle base spring and then forcefully pressed. The liver tissue will be cut into the groove of the internal cutting needle. After the surgery, the puncture site of the goat was hemostatic and disinfected, and the sampling gun was adjusted so that the liver sample was exposed in the groove of the cutting needle. The cut sample was put into the freezing tube with sterile tweezers, labeled, and preserved in liquid nitrogen.

### 2.4. Sample Processing

The soil and forage samples were dried at 20–25 °C until they reached constant weight, and then crushed, then passed through a fine sieve with a diameter of 0.075 mm, 0.5 g of the sample was taken and put into a digestion tube, a 6:1 mixture of nitric acid (HNO_3_) and hydrogen peroxide (H_2_O_2_) was added, shaken well and left to stand for 10 min after the microwave digestion procedure. Finally, the processed samples were transferred to a 100 mL volumetric flask, diluted to the graduated line, and labeled to wait for the next test. The blood and liver samples were also air-dried at 20–25 °C to reach constant weight, and 5 mL of HNO_3_, 2 mL of perchloric acid (HClO_4_), 5 mL of hydrofluoric acid (HF) were mixed, followed by microwave heating to dissolve the sample, and finally labeled for testing.

### 2.5. Sample Analysis

Inductively Coupled Plasma Atomic Emission Spectroscopy (ICP-AES) was used to analyze manganese (Mn), zinc (Zn), cobalt (Co), phosphorus (P), Cu, Mo, selenium (Se) and S content in the soil, forage, blood and liver samples. During sampling and experimentation, environmental factors such as atmospheric deposition can produce metal contamination; in order to avoid errors caused by external contamination, thus ensuring the accuracy of experimental results, the samples were subjected to laboratory quality assurance and control (QA/QC) procedures using National Soil Standard GBW07452 (GSS-23). The peak recoveries of minerals ranged from 88~107% at different concentration ranges. The detailed recoveries of each mineral were as follows: Mn (96–102%), Zn (98–107%), Co (92–104%), Cu (93–104%), P (94–105%), Mo (91–103%), Se (88–101%). The detection limits (MDLs) of the selected elements were 0.006–3.000 mg/kg, and the relative standard deviations (RSDs) of the duplicate samples were 5–29%.

Automatic Blood Cell Analyzer (Sysmex Poch-100i Veterinary, Sysmex Corporation, Shanghai, China) was used to analyze hemoglobin (Hb), red blood cell count (RBC), hematocrit value (HCT), mean corpuscular volume (MCV), mean corpuscular hemoglobin (MCH), mean corpuscular hemoglobin concentration (MCHC), and white blood cell (WBC). 

An Automatic Biochemical Analyzer (Mindraybs-420, Mindray Biotechnology Co., Ltd., Shanghai, China) was used to analyze lactate dehydrogenase (LDH), aspartate aminotransferase (AST), γ-glutamyl transpeptidase (γ-GT), ceruloplasmin (Cp), alkaline phosphatase (AKP), immunoglobulin M (IgM), immunoglobulin G (IgG), glutathione peroxidase (GSH-Px), malondialdehyde (MDA), superoxide dismutase (SOD), total antioxidant capacity (T-AOC), and Catalase (CAT).

A Hemorheology Visual Detection Analyzer (MC-FAN RH300, Tokyo, Japan) and Automated Erythrocyte Sedimentation Rate Analyzer (ALIFAX Test 1, Polverara, Italy) were used to analyze whole blood with high shear viscosity (HS), whole blood with middle shear viscosity (MS), whole blood with low shear viscosity (LS), plasma viscosity (PV), erythrocyte rigidity index (IR), erythrocyte aggregation index (EAI), and erythrocyte deformation index (TK).

### 2.6. Data Analysis

Statistical Package for the Social Science (SPSS, version 23.0, Inc., Chicago, IL, USA) was used to analyze the results. It was expressed by the mean ($\bar{x}$) ± standard deviation (SD). Differences between the two groups were initially analyzed using ANOVA, and subsequently, the independent sample *t*-tests were employed to compare specific group means, where *p* < 0.01 was a very significant difference.

## 3. Results

### 3.1. Mineral Content in the Soil and Pasture

In the soil, the Se content in the experimental pasture was notably lower at 0.02 μg·g^−1^ compared to the control pasture’s 0.06 μg·g^−1^, while there were no significant differences in the contents of Cu and S. Regarding forage, the Se content was 0.03 μg·g^−1^ in the experimental pasture and 0.07 μg·g^−1^ in the control pasture, with the S contents of 0.30% and 0.18%, respectively. The content of Se in the soil and forage from the experimental pasture was very significantly lower than that of the control pasture (*p* < 0.01), the content of S higher than that in the control pasture (*p* < 0.01), and there was no significant difference in other mineral elements, as shown in Table 1.

### 3.2. Mineral Elements in the Blood and Liver

In the blood, the Se content was 0.01 μg·g^−1^ in the experimental group and 0.1 μg·g^−1^ in the control group, while the Cu contents were 0.37 μg·g^−1^ and 1.49 μg·g^−1^, respectively. The S contents were 3.67% and 1.01%, respectively, with no significant differences in other mineral elements observed. In the liver, the Se contents were 0.1 μg·g^−1^ and 0.61 μg·g^−1^ in the experimental and control groups, respectively. The Cu contents were 50.67 μg·g^−1^ and 87.55 μg·g^−1^, respectively, while the S contents were 3.51% and 0.89%, respectively. The content of Se and Cu in the blood and liver from the experimental group were very significantly lower than those from the control group (*p* < 0.01), S was very significantly higher than that of the control group (*p* < 0.01), and there was no significant difference in other mineral elements, as shown in Table 2.

### 3.3. Main Hematological Values

In the experimental group, the levels of Hb, HCT, MCV, and MCH were 95.85 g/L, 34.94%, 50.7 fl, and 13.89 pg, respectively, compared to 133.84 g/L, 55.77%, 80.47 fl, and 19.28 pg, respectively, in the control group. The Hb, HCT, MCV, and MCH levels in the experimental group were significantly lower than those in the control group (*p* < 0.01). There were no significant differences in other indexes, as shown in Table 3.

### 3.4. Main Biochemical Parameters

The levels of Cp, IgM, and IgG in the experimental group were 2.34 IU/L, 2.24 g/L, and 6.64 g/L, respectively, while those in the control group were 5.1 IU/L, 3.11 g/L, and 10.13 g/L, respectively. The levels of Cp, IgM and IgG in the experimental group were very significantly lower than those in the control group (*p* < 0.01). Still, no significant differences were found in other indexes (Table 4).

### 3.5. Main Antioxidant Parameters

The levels of T-AOC, SOD, and GSH-Px in the experimental group were 20.13 IU/L, 47.39 IU/L and 3.97 IU/L, respectively, while those in the control group were 52.31 IU/L, 71.42 IU/L and 14.99 IU/L, respectively. The levels of T-AOC, GSH-Px, and SOD in the experimental group were very significantly lower than those in the control group (*p* < 0.01), and the content of MDA in the experimental group was 150.21 µmol/L, which was very significantly higher than that in the control group (37.17 µmol/L) (*p* < 0.01). There were no significant differences in other indicators, as shown in Table 5.

### 3.6. Main Hemorheological Parameters

In the experimental group, the levels of HS, LS, IR, EAI, and TK were 6.46 mPa/s, 13.27 mPa/s, 6.27, 5.43 and 0.69, respectively, compared to 4.37 mPa/s, 11.75 mPa/s, 4.12, 4.77 and 0.44, respectively, in the control group. Additionally, the level of PV in the experimental group was 1.41 mPa/s, whereas in the control group, it measured 2.16 mPa/s. The levels of HS, LS, IR, EAI, and TK in the experimental group were significantly higher than those of the control group (*p* < 0.01), and PV was very significantly lower than that of the control group (*p* < 0.01), as shown in Table 6.

### 3.7. Changes of the Blood Mineral Content in Yudong Black Goats after Treatment Experiment

All Yudong black goats fed CuSO_4_ solution showed recovery of Cu deficiency symptoms, which were mainly in the form of alleviation of anemia, increase in feed intake and recovery of spirit; the blood Cu level was significantly increased from 0.37 μg·g^−1^ to 1.25 μg·g^−1^ (*p* < 0.01), the other elements contents were not significantly changed, and the blood Cu level of black goats not fed with CuSO_4_ solution was not significantly changed, the content of other elements did not change significantly, as shown in Table 7.

## 4. Discussion

### 4.1. Mineral Content in the Soil and Pasture

Youyang County, Chongqing Municipality, is one of the main production areas in the Yudong black goat distribution area, and local farmers raise Yudong black goats mostly in the form of free-range, centralized rearing or a combination of both. Mineral elements play an important role in the growth, development, reproduction and evolution of domestic and wild animals [13,14,15]. Se is a non-metallic element that plays a vital role in all animals, especially ruminants [16,17,18]. The main sources of Se affecting the intake of grazing livestock are soil and forage, so the content of Se in the soil and forage is extremely important. In this study, the Se content of the soil and mixed forage in the experimental pasture was very significantly lower than that in the control pasture (*p* < 0.01), with only 0.02 μg·g^−1^ DM of soil Se and only 0.03 μg·g^−1^ DM of mixed forage Se. According to studies, normally, the elemental content of Se in the soil ranges from 0.1 to 0.7 μg·g^−1^ DM [19], and the average content of Se in the soil of China was 0.296 μg·g^−1^ DM, and the Se content in the forage is closely and positively correlated with the Se content in the soil. The Se content in the soil and forage below 0.1 μg·g^−1^ DM is considered a Se deficiency [20]. In addition, the Se content in the soil and forage below 0.040 μg·g^−1^ DM and 0.050 μg·g^−1^ DM, respectively, is considered to be severe Se deficiency. The total Se content in the soil and forage reached 0.04–0.08 μg·g^−1^ DM, and 0.05–0.1 μg·g^−1^ DM is the minimum Se content to ensure the normal growth of ruminants. Therefore, the area where this experimental pasture is located is a severe Se deficiency area.

There is a competitive antagonism between selenate and sulfate uptake in plants. Because Se and S belong to the same family of elements and are structurally similar, selenate and sulfate have the same uptake sites in the cell walls of plant roots and have similar affinities for the uptake sites. Therefore, the lower the Se content in the soil, the more S uptake by the forage. In our study, the S content of the Se-deficient experimental pasture was very significant (*p* < 0.01) higher than that of the control pasture, which is consistent with this theory.

In the natural grassland grazing ecosystem, including the soil, forage, and animals, the forage mainly obtains nutrients from the soil through its roots. The herbivores, in turn, obtain the nutrients they need by feeding on the forage [21]. Therefore, from Table 2, it can be seen that affected by low Se and high S forage, the mineral elements in Yudong black goats of the experimental group also showed low Se and high S results, and the blood and liver Se of Yudong black goats in the experimental pasture were very significantly lower than those of the control group (*p* < 0.01). The S was significantly higher than the control group’s (*p* < 0.01).

For ruminants, the ability of forage to satisfy the Cu needs of herbivores depends very much on their ability to absorb Cu [22]. After ruminants consume high S forage, excessive S will combine with Mo to form sulfomolybdate in the rumen of ruminants, and sulfomolybdate affects Cu absorption everywhere in the body [23]; in the intestinal tract, sulfomolybdate closes the absorption site of Cu and reduces the intestinal absorption of Cu, and in the bloodstream, sulfomolybdate will form a stable Cu-S-Mo protein complex with Cu, which reduces the utilization of Cu by the organism. In the liver, sulfomolybdate can directly strip Cu from metallothionein; the stripped Cu becomes a small molecule discharged through the blood and bile, and the metallothionein is filled by other Cu, which reduces the body’s absorption and utilization of Cu week after week and induces the body to have a Cu deficiency [9]. Normally, a Cu content of 7–8 μg·g^−1^ DM in forage can meet the needs of ruminants [24], but if ruminant animals live in a high Mo or high S environment, they need a higher Cu content to meet their needs [25]. In our study, the blood Cu content of the experimental group was 0.37 μg·g^−1^ DM, which was significantly lower than that of the control group (*p* < 0.01). Mean blood Cu values less than 0.50 μg·g^−1^ DM as a marker of severe Cu deficiency in ruminants. Therefore, the Yudong black goats in the experimental group had secondary Cu deficiency caused by feeding on high S forage.

### 4.2. Influence of Mineral Change in Yudong Black Goats

Hematological values can be used as essential markers to determine the degree of anemia in animals [26]. The results of our study showed that the Hb, HCT, MCV and MCH of Yudong black goats in the experimental pasture were very significantly reduced (*p* < 0.01), the significant decrease in Hb and HCT values indicated the presence of anemia indicators in Yudong black goats, and the significant decrease in MCV and MCH indicated that the type of anemia in Yudong black goats was microcytic anemia. There is a significant correlation between Cu deficiency and anemia because Cu is a cofactor of synthase involved in hemoglobin [27]. Most of the Cu in the serum is in the form of Cp, which increases the iron saturation rate in ferritin, promotes the absorption, transport and utilization of Fe in the bones [28], so Cu deficiency impairs the processes described above and leads to anemia symptoms in the organism. In Shen’s study [29], Cu-deficient Merino sheep with anemic symptoms had significantly lower Cp levels than normal Merino sheep. In our study, the Cp content in the experimental group’s blood was significantly lower than that of the control group (*p* < 0.01). In the treatment experiment, the anemia symptoms of all the Yudong black goats provided with CuSO_4_ were cured, and their Cu levels rebounded significantly (*p* < 0.01), while the Yudong black goats not provided with CuSO_4_ did not recover from the symptoms and had no significant change in their Cu levels, a finding that further validates that microcytic anemia in Yudong black goats is one of the Cu deficiency symptoms caused by Cu deficiency.

Cu affects the immune function of the animal organism. In Prohaska’s study, impaired humoral-mediated immune response and reduced number of antibody-producing cells were observed in copper-deficient mice [30]. O’Dell et al. observed that the levels of CD 4+ and total T cells were considerably decreased in Cu-deficient rodents [31]. In our study, the number of IgM and IgG in the experimental group was significantly lower than in the control group (*p* < 0.01). Cu influences the production of antibodies, participates in the composition of immunoglobulins, and significantly increases the amount of serum IgM and IgG, thus enhancing the immunity of ruminants [32]. Therefore, the immunocompetence of the experimental group of secondary Cu-deficient Yudong black goats was lower than that of the control group, and their ability to protect the organism from diseases was weaker.

In addition, animals maintain the balance between oxidation and antioxidants by virtue of the antioxidant protection system, which is divided into two categories: enzymatic and non-enzymatic systems. The non-enzymatic system mainly consists of vitamins, glutathione, Mn, Fe, Se and Cu. The enzymatic system primarily consists of antioxidant enzymes such as CAT, GSH-Px, SOD, etc [33]. The antioxidant system of animals is to stabilize and neutralize free radicals, and SOD effectively converts oxygen radicals into H_2_O_2_ and is scavenged by GSH-Px and CAT [34] and the role of GSH-Px is to reduce lipid hydroperoxides to the corresponding alcohols, and to reduce free H_2_O_2_ to water. Se and Cu are the important components of GSH-Px, and Cu is an essential component of SOD, and the levels of both of them significantly influence GSH-Px and SOD activity [35,36]. MDA is the product of lipid peroxidation, and its level in the body can directly reflect the degree of lipid oxidative damage. T-AOC is a comprehensive indicator of the body’s antioxidant function, demonstrating the body’s resistance to external stimuli and metabolism of free radicals, and the decline of the T-AOC defense system can cause a series of diseases [37,38,39]. The GSH-Px and SOD activities of the experimental group were very significantly lower than those of the control group (*p* < 0.01), indicating that Se deficiency-induced Cu secondary deficiency has reduced the activities of GSH-Px and SOD. Therefore, the body cannot scavenge away excess free radicals in the body, resulting in an imbalance between oxidative and antioxidative functions in Yudong black goats. The T-AOC activity of the experimental group was very significantly lower than that of the control group (*p* < 0.01), and the MDA was considerably higher than that of the control group (*p* < 0.01), indicating that Se deficiency-induced secondary Cu deficiency has caused severe damage to the antioxidant system of Yudong black goats.

Hemorheology mainly studies the properties of fluidity, adhesion, deformability and aggregation of red blood cells, reflecting the blood circulation condition of the animal organism [40]; under the condition of a specific shear rate range, the higher the shear rate, the lower the blood viscosity value (BV). The BV is primarily affected by the EAI and TK, the LS is primarily affected by EAI, and HS is primarily affected by erythrocyte deformation index (TK) [41] when subjected to low shear rate; erythrocytes produce aggregation and change with HCT thus producing higher BV when subjected to high shear rate, erythrocytes produce deformation and increase BV with little effect of HCT. The results of this study showed that under the condition of low shear rate, the LS of Yudong black goats in the experimental group was very significantly higher than that of the control group (*p* < 0.01), indicating that it was due to the change of EAI that led to the increase in BV in Yudong black goats. HS of Yudong black goats in the experimental group was very significantly higher than that of the control group (*p* < 0.01), indicating that erythrocytes of Yudong black goats in the experimental group produced deformation, and the BV was increased with the increase in TK. The PV of the experimental group was significantly lower than that of the control group (*p* < 0.01), indicating that the plasma proteins of Yudong black goats were abnormal and that the change of plasma proteins was also one of the factors causing the change of BV. The deformability of erythrocytes can be reflected by the TK and IR [42], TK and IR of the experimental group were very significantly higher than those of the control group (*p* < 0.01), indicating that the structure of erythrocytes of Yudong black goats in the experimental group was changed and that the oxidative stress caused by the Cu-deficient environment probably caused changes in erythrocyte membranes and the nature of the hemoglobin in the membrane, which was in accordance with the above results of the test results of the decline in the activities of GSH-Px and SOD triggered the damage of the antioxidant system of the Yudong black goats. The above results indicate that EAI, TK and IR increased in Yudong goats in the experimental group, and plasma proteins were abnormal in Yudong black goats, which together triggered an increase in BV in Yudong black goats, leading to a decrease in blood rheology, which might also be one of the reasons for the anemia symptoms of Yudong black goats.

### 4.3. Cu Nutrition for Cu-Deficient Yudong Black Goats

Since Cu deficiency can cause many diseases in goats, it is necessary to supplement Cu-deficient goats with appropriate Cu nutrients. Some scholars have reported that if Cu-deficient sheep are supplemented with 25–30 mg·kg^−1^ of CuSO_4_ or 60 mg·kg^−1^ of 1% CuSO_4_ solution every week, the sheep can be significantly improved or recovered in a fortnight [43]. In Wang’s [44] experiment, Cu deficiency symptoms completely disappeared after 7 d of oral administration of 10 mg·kg^−1^ of CuSO_4_ to Cu-deficient crossbred diseased sheep. In Apache’s [45] test, after daily oral administration of 10 mg·kg^−1^ 1% CuSO_4_ solution to Cu-deficient crossbred little-tailed chilly sheep crossbred sheep, Cu deficiency symptoms were completely recovered after 3 weeks. In our study, after we provided 60 mg·kg^−1^ of 1% CuSO_4_ solution to Yudong black goats for 20 d, all the black goats recovered their health, and there was a very significant enhancement of blood Cu in their bodies (*p* < 0.01); therefore, the symptoms of anemia and loss of appetite in Yudong black goats of the experimental group were Cu deficiency symptoms, and supplementing Cu deficient Yudong black goats with the appropriate amount of CuSO_4_ could alleviate the Cu deficiency symptoms.

## 5. Conclusions

In summary, through a series of studies, our research findings align with initial hypotheses; the Cu deficiency in Yudong black goats was a secondary Cu deficiency caused by Se deficiency: soil Se deficiency induced an increase in S uptake by forage, and Yudong black goats feeding on high S forage reduced their own Cu uptake and utilization, resulting in secondary Cu deficiency, which was mainly manifested as anemia, decreased immune function, impaired antioxidant system, and reduced blood rheology in Yudong black goats. Furthermore, Cu deficiency symptoms can be alleviated by providing appropriate amounts of CuSO_4_ solution.

## Figures and Tables

**Table 1:** The mineral content in the soil and forage (μg·g^−1^).

Elements	Soil			Forage		
	Experimental Pasture	Control Pasture	*p*-Value	Experimental Pasture	Control Pasture	*p*-Value
Mn	99.45 ± 18.7	107.95 ± 22.95	0.24	57.64 ± 10.12	57.74 ± 11.15	0.92
Zn	29.89 ± 4.35	29.95 ± 4.48	0.89	6.61 ± 1.81	6.94 ± 1.67	0.24
Co	5.20 ± 1.03	6.06 ± 1.15	0.11	4.07 ± 0.99	3.71 ± 1.08	0.17
Cu	48.25 ± 16.83	46.89 ± 10.54	0.76	10.14 ± 1.96	9.97 ± 2.01	0.90
P	44.94 ± 5.41	45.81 ± 6.21	0.75	353.60 ± 49.31	356.15 ± 48.45	0.93
Mo	2.49 ± 0.43	2.43 ± 0.47	0.88	1.81 ± 0.33	1.79 ± 0.23	0.91
Se	0.02 ± 0.001 ^B^	0.06 ± 0.003 ^A^	<0.01	0.03 ± 0.002 ^B^	0.07 ± 0.003 ^A^	<0.01
S (%)	0.39 ± 0.11	0.37 ± 0.10	0.27	0.30 ± 0.01 ^A^	0.18 ± 0.01 ^B^	<0.01

Note: Means in the same row with different superscripts ^A^, ^B^ are significantly different, with *p* < 0.01. Mn—manganese; Zn—zinc; Co—cobalt; Cu—copper; P—phosphorus; Mo—molybdenum; Se—Selenium; S—sulfur.

**Table 2:** The mineral content of the blood and liver in Yudong black goats (μg·g^−1^).

Elements	Blood			Liver		
	Experimental Group	Control Group	*p*-Value	Experimental Group	Control Group	*p*-Value
Mn	0.71 ± 0.15	0.69 ± 0.14	0.34	3.89 ± 1.10	3.89 ± 1.13	0.91
Zn	2.99 ± 0.14	3.09 ± 0.09	0.67	44.94 ± 9.95	45.19 ± 9.65	0.89
Co	0.45 ± 0.10	0.44 ± 0.18	0.72	5.66 ± 1.08	5.75 ± 0.99	0.76
Cu	0.37 ± 0.23 ^B^	1.49 ± 0.25 ^A^	<0.01	50.67 ± 11.90 ^B^	87.55 ± 9.35 ^A^	<0.01
P	198.05 ± 17.85	199.75 ± 22.95	0.93	541.45 ± 31.45	527.85 ± 29.75	0.89
Mo	0.15 ± 0.03	0.15 ± 0.03	0.95	1.28 ± 0.09	1.43 ± 0.23	0.14
Se	0.01 ± 0.005 ^B^	0.10 ± 0.06 ^A^	<0.01	0.10 ± 0.03 ^B^	0.61 ± 0.06 ^A^	<0.01
S (%)	3.67 ± 0.30 ^A^	1.01 ± 0.44 ^B^	<0.01	3.51 ± 0.39 ^A^	0.89 ± 0.72 ^B^	<0.01

Note: Means in the same row with different superscripts ^A^, ^B^ are very significantly different, with *p* < 0.01. Mn—manganese; Zn—zinc; Co—cobalt; Cu—copper; P—phosphorus; Mo—molybdenum; Se—Selenium; S—sulfur.

**Table 3:** Hematological values in Yudong black goats.

Hematological Values	Experimental Group	Control Group	*p*-Value
Hb (g/L)	95.85 ± 9.63 ^B^	133.84 ± 11.69 ^A^	<0.01
RBC (10^12^/L)	5.86 ± 0.19	5.89 ± 0.20	0.91
HCT (%)	34.94 ± 2.65 ^B^	55.77 ± 2.99 ^A^	<0.01
MCV (fl)	50.70 ± 3.59 ^B^	80.47 ± 6.74 ^A^	<0.01
MCH (pg)	13.89 ± 2.34 ^B^	19.28 ± 2.18 ^A^	<0.01
MCHC (%)	19.78 ± 1.93	20.37 ± 1.84	0.34
WBC (10^9^/L)	8.05 ± 0.60	8.14 ± 0.57	0.88

Note: Means in the same row with different superscripts ^A^, ^B^ are significantly different, with *p* < 0.01. Hb—hemoglobin; RBC—red blood cell; HCT—hematocrit; MCV—mean corpuscular volume; MCH—mean corpuscular hemoglobin; MCHC—mean corpuscular hemoglobin concentration; WBC—white blood cell.

**Table 4:** Biochemical values in the blood of Yudong black goats.

Biochemical Values	Experimental Group	Control Group	*p*-Value
LDH (µmol/L)	4.05 ± 0.60	4.11 ± 0.527	0.89
AST (IU/L)	22.07 ± 4.78	22.72 ± 4.43	0.24
γ-GT (IU/L)	15.96 ± 3.09	15.94 ± 2.69	0.93
Cp (IU/L)	2.34 ± 0.64 ^B^	5.10 ± 0.73 ^A^	<0.01
AKP (IU/L)	216.75 ± 11.05	221.85 ± 11.05	0.78
IgM (g/L)	2.24 ± 0.23 ^B^	3.11 ± 0.24 ^A^	<0.01
IgG (g/L)	6.64 ± 0.49 ^B^	10.13 ± 0.71 ^A^	<0.01

Note: Means in the same row with different superscripts ^A^, ^B^ are very significantly different, with *p* < 0.01. LDH—lactate dehydrogenase; AST—aspartate aminotransferase; γ-GT—γ-glutamyl transpeptidase; Cp—ceruloplasmin; AKP—alkaline phosphatase; IgM—immunoglobulin M; IgG—immunoglobulin G.

**Table 5:** Blood antioxidant indicators in the blood of Yudong black goats.

Antioxidant Values	Experimental Group	Control Group	*p*-Value
T-AOC (IU/L)	20.13 ± 2.06 ^B^	52.31 ± 6.59 ^A^	<0.01
SOD (IU/L)	47.39 ± 1.88 ^B^	71.42 ± 7.06 ^A^	<0.01
GSH-Px (IU/L)	3.97 ± 1.47 ^B^	14.99 ± 2.28 ^A^	<0.01
MDA (µmol/L)	150.21 ± 16.72 ^A^	37.17 ± 8.21 ^B^	<0.01
CAT (µmol/L)	271.15 ± 28.90	264.35 ± 32.30	0.73

Note: Means in the same row with different superscripts ^A^, ^B^ are significantly different, with *p* < 0.01. T-AOC—total antioxidant capacity; SOD—superoxide dismutase; GSH-Px—glutathione peroxidase; MDA—malondialdehyde; CAT—catalase.

**Table 6:** Hemorheology indexes of Yudong black goats.

Hematological Values	Experimental Group	Control Group	*p*-Value
HS (mPa/s)	6.46 ± 0.32 ^A^	4.37 ± 0.41 ^B^	<0.01
MS (mPa/s)	6.15 ± 0.39	5.39 ± 0.49	0.25
LS (mPa/s)	13.27 ± 0.72 ^A^	11.75 ± 0.37 ^B^	<0.01
PV (mPa/s)	1.41 ± 0.19 ^B^	2.16 ± 0.47 ^A^	<0.01
IR	6.27 ± 1.06 ^A^	4.12 ± 0.94 ^B^	<0.01
EAI	5.43 ± 0.20 ^A^	4.77 ± 0.36 ^B^	<0.01
TK	0.69 ± 0.08 ^A^	0.44 ± 0.09 ^B^	<0.01

Note: Means in the same row with different superscripts ^A^, ^B^ are very significantly different, with *p* < 0.01. HS—whole blood with high shear viscosity; MS—whole blood with middle shear viscosity; LS—whole blood with low shear viscosity; PV—plasma viscosity; IR—erythrocyte rigidity index; EAI—erythrocyte aggregation index; TK—erythrocyte deformation index.

**Table 7:** Effect of feeding CuSO_4_ on blood minerals in Yudong black goats (μg·g^−1^).

Elements	Supplying CuSO_4_			Without Supplying CuSO_4_		
	0d	10d	20d	0d	10d	20d
Mn	0.70 ± 0.13	0.71 ± 0.12	0.71 ± 0.18	0.71 ± 0.17	0.70 ± 0.16	0.70 ± 0.14
Zn	2.99 ± 0.15	2.99 ± 0.12	2.98 ± 0.16	2.99 ± 0.09	3.00 ± 0.09	2.99 ± 0.11
Co	0.45 ± 0.11	0.44 ± 0.14	0.45 ± 0.09	0.45 ± 0.12	0.45 ± 0.19	0.45 ± 0.14
Cu	0.37 ± 0.23 ^B^	0.62 ± 0.21 ^A^	1.25 ± 0.30 ^A^	0.37 ± 0.25	0.38 ± 0.23	0.37 ± 0.24
P	198.17 ± 17.90	198.05 ± 12.62	199.05 ± 20.03	199.65 ± 20.95	199.43 ± 20.65	198.91 ± 19.01
Mo	0.15 ± 0.03	0.15 ± 0.02	0.15 ± 0.02	0.15 ± 0.02	0.15 ± 0.03	0.15 ± 0.04
Se	0.01 ± 0.005	0.01 ± 0.009	0.01 ± 0.008	0.01 ± 0.004	0.01 ± 0.005	0.01 ± 0.007
S (%)	1.01 ± 0.44	1.02 ± 0.52	1.01 ± 0.37	1.01 ± 0.44	1.01 ± 0.32	1.00 ± 0.49

Note: Means in the same row with different superscripts ^A^, ^B^ are very significantly different, with *p* < 0.01. Mn—manganese; Zn—zinc; Co—cobalt; Cu—copper; P—phosphorus; Mo—molybdenum; Se—Selenium; S—sulfur.

## Data Availability

Data are provided upon request due to privacy restrictions. The data provided in this study are available upon request from the corresponding author. As this paper is part of a series of studies, the data are not publicly available.
